# Italian law n. 219/2017 on consent and advance directives: survey among Ethics Committees on their involvement and possible role

**DOI:** 10.1186/s12910-022-00858-w

**Published:** 2022-11-16

**Authors:** Corinna Porteri, Giulia Ienco, Edda Mariaelisa Turla, Carlo Petrini, Patrizio Pasqualetti

**Affiliations:** 1 Bioethics Unit, IRCCS Istituto Centro San Giovanni di Dio Fatebenefratelli, Via Pilastroni, 4, 25125 Brescia, Italy; 2 Bioethics Unit, Istituto Superiore di Sanità, Rome, Italy; 3 Department of Public Health and Infectious Diseases, Section of Medical Statistics, Sapienza Università di Roma, Rome, Italy

**Keywords:** Law n. 219/2017, Ethics committees, Informed consent, Shared care planning, Advance directives, Training, Bioethics, Clinical ethics

## Abstract

**Background:**

On December 2017 the Italian Parliament approved law n. 219/2017 “Provisions for informed consent and advance directives” regarding challenging legal and bioethical issues related to healthcare decisions and end-of-life choices. The law does not contain an explicit reference to Ethics Committees (ECs), but they could still play a role in implementing the law.

**Methods:**

A questionnaire-based survey was performed among the ECs of the Italian Institute for Research and Care belonging to the Network of neuroscience and neurorehabilitation, with the aim of (1) knowing whether the ECs participated and, if so, how in the process of implementation of law n. 219/2017 in the referring institutes; (2) investigating the point of view of the ECs regarding their possible involvement in the process; (3) exploring the contribution ECs can provide to give effective implementation to the law principles and provisions.

**Results:**

Seventeen ECs out of thirty took part in the survey; the characteristics of the responding and non-responding committees are similar, so the responding ECs can be regarded as representative of all ECs in the Network. Nine ECs did not discuss the law in anyway: the main reason for this is that the referring institutions (6) and the health care professionals (3) did not ask for an EC intervention. Nevertheless, the large majority of the ECs believe that their involvement in the implementation of the law as a whole is appropriate (8) or absolutely appropriate (6), while 3 of them are neutral. No EC believes that the involvement is inappropriate. The aspect of the law on which the 14 ECs converge in considering the EC involvement appropriate/absolutely appropriate is the one related to the health facilities obligation to guarantee the full and proper implementation of the principles of the law.

**Conclusions:**

Our survey confirms that ECs believe they can play a role in the implementation of law n. 219/2017, although this does not entirely correspond to what the committees have actually done in reality. This role could be better exercised by ECs specifically established for clinical practice, which would have a composition, functioning and a mandate better suited to the purpose. This supports the call for a national regulation of ECs for clinical practice.

**Supplementary Information:**

The online version contains supplementary material available at 10.1186/s12910-022-00858-w.

## Introduction

On 22 of December 2017 the Italian Parliament finally approved law n. 219/2017 “Provisions for informed consent and advance directives” regarding challenging legal and bioethical issues related to healthcare decisions and end-of-life choices [1]. The law arose from almost 30 years of a fervent and heated political and public debate concerning end-of-life topics, consent to medical treatment and the meaning of healthcare, and passed after many legislative initiatives that never became law [2, 3]. The law concerns issues on which there are different and even conflicting moral beliefs and cultural approaches, such as human dignity, self-determination in healthcare and its possible limits, inviolability or availability of human life, the role of the physician in front of the patient’s choices, the proper ends of medicine [4, 5].

The social discussion on the matter was also influenced by the jurisprudential elaboration originated from well-known cases faced by the Italian courtrooms (as Piergiorgio Welby, Eluana Englaro and Fabiano Antoniani). These cases highlighted the importance—and the persistent lack in the Italian legislative corpus—of a comprehensive discipline about end-of-life care, in a context of never-stopping advancements of medicine and evolution of population’s health state and life expectancies [6, 7]. Furthermore, the law responds to the need for implementing the international and supranational regulatory framework, such as the Oviedo Convention on Human Rights and Biomedicine (art. 28), the European Convention on Human Rights (ECHR, art. 2–3) and the Charter of Fundamental Rights of the European Union (CFR art. 1–2-3) [8, 9].

The provisions contained in law n. 219/2017 are based on the fundamental principles concerning the human being rooted in the Italian Constitution, expressly mentioned in the first article of the law, and aim to protect the person’s rights to life, health, dignity and self-determination in every moment of the person’s life, even when the individual is temporarily or no longer able to decide and express choices about health-care [10, 11].

The law promotes the person’s autonomy, and a patient-physician relationship shifted to a patient-centred approach [12–14]: the person's view, preferences and wills are valued, and the goal of the therapeutic relationship appears to be the pursuit of the patient's health, understood as the best physical, psychological and relational well-being achievable by the person taking into consideration both medical criteria and the person’s individuality [15, 16].

The discipline drawn up by the law to achieve such patient-physician relationship is basically composed by three elements: Informed consent (article 1), Shared care planning (article 5) and Advance directives (article 4) (in the Italian text respectively “Consenso informato”, “Pianificazione condivisa delle cure” and “Disposizioni anticipate di trattamento”). The law also considers pain therapy, prohibition of unreasonable obstinacy in treatment and dignity in the end of life (article 2); and the situation of minors and incapacitated adults (article 3).

As a brief illustration of the law content, article 1 (Informed consent) provides for the patient’s rights to be fully informed about his/her health conditions (or, on the contrary, not to be informed and to delegate health-related decisions), to give consent or dissent to medical treatments, to withhold consent to unwanted therapies, even if life-sustaining. Article 2 (Pain therapy, prohibition of unreasonable obstinacy in treatment and dignity in the end of life) ensures the patient’s right to an appropriate pain therapy and establishes the physician’s duty to refrain from unreasonable obstinacy and unnecessary or disproportionate treatment in end-of-life care. Article 3 (Minors and incapacitated adults) contains specific rules for minors and incapacitated adults on informed consent and decision-making and establishes these persons’ right to the valuing of their capacity of understanding and making choices. Article 4 (Advance directives) ensures the citizen’s right to express wills about medical treatments and give directions of care in anticipation of a possible future inability to self-determination. Article 5 (Shared care planning) states that in case of a chronic and disabling disease or disease characterized by an inevitable progression with poor prognosis, patient and physician can discuss and prepare together a care plan which health providers are obliged to abide in the event the patient becomes unable to express consent or reaches a condition of incapacity. To refer to this process, the expression "advance care planning" is normally used in international language. The Italian wording emphasizes the sharing of the process. Rules established about advance directives and shared care planning also include the person’s right to nominate a trusted person with power of representation in the relationship with healthcare professionals and organizations. Article 1 also provides for the health facilities obligation to guarantee, through their own organizational modalities, the full and proper implementation of the principles of the law, ensuring the necessary information to patients and adequate training of staff on care relationship, pain therapy and palliative care.

In this paper we consider attitudes and practices about the law provisions from the perspective of the Ethics Committees [ECs].

ECs are independent, interdisciplinary bodies that are regulated differently in different countries. When established at local level, their main functions are the evaluation of research protocols, consultation on ethical issues related to daily clinical practice and bioethics training. Based on different national or local regulations, these functions may be performed by the same body or by distinct committees [17]. In Italy ECs “are responsible for ensuring the protection of the rights, safety and well-being of persons undergoing clinical trials and for providing a public guarantee of such protection”. Moreover, “where not already assigned to specific bodies, the ethics committees can also carry out consultative functions in relation to ethical issues connected with scientific and welfare activities, in order to protect and promote the values ​​of the person. Furthermore, the ethics committees can propose training initiatives for health professionals in relation to issues relating to bioethics” [18].

Law n. 219/2017 does not contain an explicit reference to ECs and thus does not consider their potential involvement in the implementation of its content. Nevertheless, some Authors either highlighted the shortcoming [19] or recalled the possible contribution of the ECs in the enforcement of the law [5]. In fact, as the law touches on principles and human values in medicine, the ECs, that are a space for ethical reflection and deliberation, may be an actor in the process of the law implementation. Experiences of international ECs, as the Comités de Ética Asistencial in Spain, have already showed that ECs may have a relevant role in promoting the principles of informed consent and advance directives, that may also include advance care planning, regulated in the Spanish law n. 41/2002 [20].

While few surveys were conducted on law n. 219/2017 involving citizens [21] and health care professionals [6, 14, 22–24] we are not aware of any research targeting ECs.

Few years after the entry into force of the Italian law, on January 31, 2018, this survey aimed at (1) knowing whether the ECs participated and, if so, how in the process of implementation of law n. 219/2017 in the referring institutes; (2) investigating the point of view of the ECs regarding their possible involvement in the process; (3) exploring the contribution ECs can provide to give effective implementation to the law principles and provisions. Based on the survey results an additional aim of the study was to discuss the roles of ECs.

## Methods

### Survey

A questionnaire-based survey was performed among the ECs of the Italian Institute for Research and Care (IRCCS) belonging to the IRCCS Network of neuroscience and neurorehabilitation.

A letter of invitation containing a brief illustration of the study scope and design was sent by email to the chiefs of the ECs with the request to participate in the research by filling in an online questionnaire. The questionnaire could be completed either by the EC chief or by his/her delegate, preferably with expertise in bioethics or bio-law.

The first invitation was sent at the end of June 2021 and, following reminder, the survey was completed in October 2021.

The present study was favourably reviewed by the IRCCS Fatebenefratelli EC (opinion n. 44/2021).

### Questionnaire

A semi-structured questionnaire consisting of a total of 34 questions—19 closed, 11 open-ended, and 4 only consisting of a request to provide the link to available documentation—was developed by two of the Authors (CPo and PP). Depending on the answer, a number of questions allow some of the subsequent questions to be skipped.

The questionnaire was organized into the following areas: ECs participation in the discussion/implementation of the law; ECs production and/or revision of documents in the light of the new law; opinion on the involvement of the ECs in the implementation of the law as a whole and of each article; possible ECs contribution on the implementation of each article of the law. One final question asked for the clinical condition, within the field of neuroscience, for which the involvement of the ECs in the implementation of the law could be most useful.

Essential information regarding the ECs taking part in the survey was also collected.

The initial version of the questionnaire was submitted for revision to three reviewers with expertise in bioethics and bio-law and experience as members of ECs to collect their comments on the content and formulation of the questions.

The revised questionnaire was transferred to an online platform and submitted to five EC members to assess the clarity of the questions and the functionality of the platform.

A final version of the online questionnaire was developed after the test and used in the survey. [The questionnaire is available as Additional file 1].

### Participants

The ECs of the IRCCS belonging to the Italian Network of neuroscience and neurorehabilitation were invited to participate. The IRCCS are recognized by the Italian Ministry of Health (MoH) as national institutes of excellence for research and care. The IRCCS Network of neuroscience and neurorehabilitation is the country’s largest research network in the field; it was founded in 2017 by the MoH to promote scientific and technological research and the cooperation among the institutes. We chose to investigate the ECs belonging to this Network as the survey promoter (IRCCS Fatebenefratelli) is part of it and we are therefore particularly interested in clinical neurological conditions, which, especially in the case of neurodegenerative diseases, may be an important target of the law.

The IRCCS Network of neuroscience and neurorehabilitation currently includes 30 IRCCS in the North, Central and South Italy; four of them refer to ECs common to other IRCCS, while other three have separate branches in different Italian Regions that refer to an EC other than that of the head institute. In total the IRCCS Network of neuroscience and neurorehabilitation refers to 30 ECs.

These ECs are set up following the Italian national regulation [18] and are included in the registry of the Italian Medicine Agency (AIFA).

### Data analysis

The target population comprised 30 ECs. In case some ECs would not accept to participate, the available sample could not be considered as randomly drawn from the whole population and thus the statistical analysis of the questionnaires could not be inferential. On the other hand, we will gather information about the eventual ECs that do not take part in this survey and descriptively compare the characteristics of the two groups.

Information was collected by means of an online platform (Google Forms) which allows to export data in a spreadsheet (Windows Excel) to perform statistical analysis. Two of the Authors (CPo and PP) executed data analysis [The dataset with responses to the questionnaire is available as Additional files 2, 3].

Since the number of ECs was small, we avoided the use of percentages and reported absolute frequencies.

## Results

Seventeen ECs out of thirty took part in the survey: 10 ECs (out of 15 invited) are placed in the North of Italy, 4 (out of 9) in the Centre, and 3 (out of 6) in the South. Nine of the seventeen ECs work not only for the institutes belonging to the Network but, with the same role, also for other health facilities. The majority of the ECs regulations (13) report all three functions of (i) research protocol evaluation, (ii) consultation, and (iii) training. In three cases the IRCCS also refers to another EC for clinical practice.

Nine of the EC members (chief or delegate) who completed the questionnaire had expertise in bioethics, 3 in clinical area, 3 in scientific area, 2 in law. The compilation of the questionnaire took from 5 to 30 min.

Nine ECs that responded to the questionnaire did not discuss the law. The main reason for this is that the referring institutions (6) and the health care professionals (3) did not ask for the EC intervention. In addition to the lack of external request, for one participant the discussion of the law did not fall within the institutional tasks of the body. Two ECs intended to discuss the law in the near future, while one left the reading of the law to the individual members. No respondent indicated lack of time as a reason for not discussing the law.

The remaining eight ECs discussed the law in different ways: through spontaneous theoretical reflection (4), in response to a request for an opinion on specific clinical cases (4), following questions posed by the reference structures (3). Four ECs discussed the law in training activities for ECs members (3) and training for health professionals organized spontaneously by the EC (1). Informed consent was the aspect of the law more frequently discussed (5), followed by pain therapy—dignity in the end of life (3), issues related to minors (3), advance care directives (3), issues related to incapacitated adults (2), shared care planning (2), and organizational and training obligations (1).

In 4 cases the law was an opportunity for the production of new documents and for the revision of existing documents produced by the ECs, although two were still being written and/or reviewed, one was not available and one consisted of a clinical case opinion. In two cases the ECs were aware that the referring facilities had drafted or revised existing documents, but this was done without the EC cooperation.

The large majority of the ECs believe that their involvement in the implementation of the law as a whole is appropriate (8) or absolutely appropriate (6), while 3 of them are neutral. No EC believes that the involvement is inappropriate. The opinion regarding the opportunity of the involvement is mixed across respondents that discussed or did not discuss the law: 8 ECs that did not discuss the law regard the involvement of the ECs as absolutely appropriate (4) or appropriate (4) and one is neutral; while two ECs that discussed the law are neutral, and the others consider the involvement as absolutely appropriate (2) or appropriate (4) [Table 1].

**Table 1:** Opinion on the involvement in the implementation of law n. 219/2017 among ECs who discussed and did not discuss the law

Involvement in the implementation of the law	ECs who discussed the law	ECs who did not discuss the law	All ECs
Absolutely inappropriate	0	0	0
Inappropriate	0	0	0
Neutral	2	1	3
Appropriate	4	4	8
Absolutely appropriate	2	4	6

The aspect of the law on which the 14 ECs converge in considering the EC involvement appropriate/absolutely appropriate is the one related to the health facilities obligation to guarantee the full and proper implementation of the principles of the law; although the degree of agreement on the EC involvement in other aspects of the law is also very high: from 10 on shared care planning, to 11 on advance directives, 12 on minors and 13 on informed consent, pain therapy—dignity in the end of life, and incapacitated adults.

Four out of the fourteen committees that consider appropriate the involvement of the ECs in the implementation of the law did not complete the section of the questionnaire relating to the type of contribution that the ECs could make.

According to the committees that responded, the EC possible contribution mainly regards the support to health facilities in organizing and teaching in training courses to fulfil the obligations required by the law (6). In addition, two ECs primarily recall the EC consultative role as advisory body for doctors, trustees and patients; while two identify a possible contribution in the development of clear indications and guidelines on the different aspects of the law. One EC mainly refers to roles of assessment and verification; one respondent believes the EC should play a central role in every aspect of the law application; and one invokes an EC proactive role in assuring that the law is known and effective within the reference structures.

In the respondents’ opinion, the clinical conditions for which the involvement of the EC could be most useful include in particular neurodegenerative diseases (as Parkinson, SLA, Huntington, dementia), but also brain injuries and disorders of consciousness.

## Discussion

The survey involved the ECs belonging to the Network of neuroscience and neurorehabilitation to which the research promoters belong. We consider that this circumstance did not cause any problems in the conduct of the study to which, however, two Authors (CPe and PP) from other institutions also contributed.

The geographical distribution of the ECs that responded to the questionnaire is not completely homogeneous across the country, with a greater number of responses observed in Northern Italy (response rate 67%) than in the South (50%) and the Centre (44%). Despite of this, the characteristics of the ECs that participated in the survey are almost comparable to those of the ECs that did not participate in terms of structures which refer to the ECs and functions attributed to the body. The AIFA registry of the Italian ECs reports that 7 out of 13 of the ECs which did not participate in the research (versus 9 out of 17 of the respondents) work not only for the institutes belonging to the Network but also for other health facilities. In addition, information retrieved from the institutional IRCCS and/or the ECs websites indicates that 10 non-responding ECs out of 13 (versus 13 out of 17 of the respondents) maintain all the three functions of research protocol evaluation, consultation, and training. In two cases (three for the respondents) the institutes also refer to another EC for the ethical issues related to clinical practice. Overall, the ECs that responded to the survey can thus be regarded as representative of all the ECs of the IRCCS Network of neuroscience and neurorehabilitation. All the ECs are also equal with respect to composition, which is defined by current Italian regulation.

The key finding of the study is the large majority (82%) of the respondents' belief that the involvement of the ECs in the implementation of the law is appropriate/ absolutely appropriate. This means that people who know the ECs from the inside and have direct experience of their work consider that these bodies can and should have a role in relation with the provisions of the law. This view does not fully correspond to what the ECs did in the reality, where in fact the majority of them did not discuss the law in any way. The discrepancy between what the ECs could and should do in the respondents’ opinion and what they have actually done so far is mainly justified by the lack of explicit request from the referring institutions and the health care professionals, which may in turn be related to the number and size of facilities referring to the committee. This seems to mean that, in general, the ECs interpret their role in terms of responding to the demands of the referring institutions, which is certainly part of their duties, rather than in terms of being proactive, which, however, is also a possibility for ECs. This attitude could be due to the fact that more than half of the ECs work for a number of health care facilities, which can make a direct relationship with institutions quite complicated; however the results are mixed, with 5 out of 9 ECs that have multiple facilities having discussed the law, in response to questions from referring facilities (3) upon request for clinical case opinion (3), in terms of spontaneous reflection (2) and in training for EC members (1). On the other hand, having been established primarily for the evaluation of clinical trials certainly makes ECs more oriented toward research than clinical practice and education: this is made clear by the respondent who, while believing that the ECs involvement in the implementation of the law is appropriate, responds that discussion of the law is not part of the EC institutional tasks.

All respondents who consider the ECs involvement in the implementation of the law appropriate/absolutely appropriate believe they should be involved in the institutions effort to ensure the full and proper implementation of the principles of the law, especially with a supporting role in organizing and teaching in training courses. This is actually a crucial contribution that the ECs can make regarding the law. Indeed, the current legislation governing the composition and operation of Italian ECs [18] requires that members have documented knowledge and multidisciplinary expertise: the ECs must include, among other components especially indicated for the evaluation of clinical trials, an expert in bioethics, an expert in law, a representative of volunteer or patient associations, a general practitioner, at least three clinicians and a representative of other health professions. These members are well placed to organize training courses or be lecturers themselves in courses that take into account the different aspects of the law: the legal, ethical, clinical ones, and the citizens’/patients’ perspective. Especially with regard to informational and educational activities, also conducted through the draft of documents and guidelines, a proactive role of the ECs would have been possible and would have led to a better alignment between the views expressed about the appropriateness of ECs involvement and the effectiveness of their activities.

The aspect of the law least indicated by the ECs for their involvement is shared care planning: this is an important provision of law n. 219/2017 that has specific relevance for neurodegenerative disorders [25]. The provision appears for the first time in a regulatory text, although a shared process of care planning was already a way of operating in best clinical practices [26, 27]. This may be due to the idea, made explicit by one respondent, that care planning is a process primarily concerned with the doctor-patient relationship. Respondents nevertheless indicated possible contributions from the EC, consisting in training, drafting of guidance, and advice on shared care planning documents or in complex situations. The same kind of EC contribution has been suggested for advance directives.

Overall, the survey responses on the one hand refer to the potential that ECs ideally possess and on the other reflect the circumstance that these bodies have multiple functions of which, however, the function of evaluating research protocols imprints all the activity. The residuality of ECs activities unrelated to protocol evaluation is confirmed by a survey on clinical ethics consultation among Italian ECs in 2016 showing that, although 72,6% of the ECs were able to provide ethics consultation by policy, ethics consultation in clinical and research practice were largely underappreciated and not well understood by users [28].

At the same time, there are examples of Italian ECs specifically dedicated to clinical practice that have worked intensively on the law, through training for health care professionals and citizens [29, 30], production of informative documents and promotion of a space for ethics consultation on advance directives [31].

A number of ECs for clinical practice have also posted on their institutional website documents and materials they have produced as a result of their work on the law, particularly with regard to shared care planning and advance directives [32–35]. These are virtuous examples that well represent what ECs can concretely do when actually attentive to the bioethical issues most important to people's health and lives, such as being able to live through illness and death according to one's own values and beliefs.

A recent survey with health care professionals working in Italy, France, Spain, and UK regarding the application of norms related to informed consent, shared care planning, advance directives, palliative care and end-of-life care confirms the need to value the role of ECs for clinical practice, which, when well trained, can be a fundamental point of reference for health professionals and support in addressing ethical issues in daily clinical practice [20].

On the contrary, the confinement of the work of ECs to the evaluation of research protocols, albeit important, is a sign of an impoverishment of the space for ethical action that these bodies should have to promote a better medicine within society.

All of this opens the discussion of whether it is appropriate or even necessary to have separate ECs for research and clinical practice to ensure that ECs can effectively perform the functions of consultation and education. With regard to our discourse, ECs consultation may for instance be related to situation of discrepancy between the view of patient or trusted person and physician, or to the condition of minors and people with poor capacity; while education could be focused on enhancing the knowledge of the law principles and the related tools offered by the norm in health care professionals and the public, and on improving the communication ability of the health-care staff.

In fact, although ECs established under Ministerial Decree 8 February 2013 [18] “can also carry out consultative functions in relation to ethical issues connected with scientific and healthcare activities, in order to protect and promote the values of the person", current Italian legislation [18, 36] attributes to ECs the function of evaluating clinical trials of medicines and medical devices. As a result, the operation, composition, organization of the committees are clearly oriented towards clinical trials, which makes these bodies less suitable for the evaluation of clinical cases and advisory activities on clinical practice. On the other hand, nowadays in Italy there is no national legislation for the establishment and regulation of ECs for clinical practice (or clinical ethics), which are therefore left to the decision of individual institutions (without, however, these committees having an official status) or to regional regulation [37, 38].

We consider that spontaneous local experiences make clear the need for regulatory intervention to regulate at national level the establishment and functioning of committees for clinical practice rooted in the territory, with appropriate multidisciplinary expertise and operating close to healthcare staff and patients. This need was also emphasized by the Italian National Bioethics Committee (CNB), which in its 2017 opinion [39] noted the marginal and tendentially residual role attributed to clinical ethics by the current Italian regulatory framework that focuses on research protocols, and proposed a distinction between ECs for clinical trials and ECs for clinical ethics. In the CNB opinion, the latter, in addition to their role in evaluating clinical cases, should have a role in bioethics education in health care facilities and in raising bioethical awareness among the general public. Training activities, which respondents to our survey indicated as an important contribution that ECs can make to the implementation of law n. 219/2017, could also find further enhancement by ECs specifically dedicated to clinical ethics. A distinction between ECs on the basis of their functions already exists in other countries, such as US, or, in Europe, UK and Spain [39]. Although both of them are interdisciplinary bodies, clinical ECs composition is more oriented towards clinical-assistance competences, which in addition to clinical staff, bioethicists, layers, patient representatives, may also include psychologists, social workers, spiritual assistants. Moreover, unlike research ECs, which give binding opinions, clinical ECs express non-binding consultative advice [40, 41].

We agree with the CNB’s call to separate the ECs on the basis of their functions, and we think that the opportunity may also be provided by the re-organization of the Italian ECs in light of EU Regulation 536/2014 on clinical trials [42], which will make even more difficult to allow these bodies a connection with the territory and the possibility of an effective space for ethical activities other than the evaluation of clinical trials [43].

### Limitations of the study

Five ECs did not provide information on their regulations, but for all of them it was possible to retrieve the missing information on the Internet.

We asked the chief of the ECs or his delegate to participate in the survey, so responses that do not describe facts are inevitably affected by the subjectivity of the respondent and do not necessarily reflect the views of the relevant EC in its collegiality. In addition, the ECs of the IRCCS belonging to the Network of neuroscience and neurorehabilitation are only a part of the Italian ECs, which, according to the AIFA registry, in 2021 numbered 91. It is possible that ECs of institutions dealing with other kinds of disease, especially cancer or other terminal illnesses where particularly pain therapy and shared care planning are more prevalent, have had more requests and/or more opportunities to discuss the law. Although we should point out that a good number of the respondent ECs also deal with more than just neurological diseases. However, a survey extended to all Italian ECs would have provided a broader picture.

Finally, looking at the possible role of ECs regarding law n. 219/2017, we collected opinions only from the ECs themselves and did not involve other stakeholders: we cannot exclude that ECs may be more prone to value their role in relation with the law provisions than other stakeholders in the regulatory system or society, which may be a barrier in the ECs involvement.

## Conclusions

Our survey confirms that ECs believe they can play a role in the implementation of law n. 219/2017, although this does not entirely correspond to what the committees have actually done in reality. This role could be better exercised by ECs specifically established for clinical practice, rather than by committees primarily set up for the evaluation of clinical trials. ECs for clinical practice would in fact have a composition, mode of functioning and, above all, a mandate better suited to the purpose of working on the primary object of the law, which is the relationship between patient and physician/medical staff. This supports the call for a national regulation of ECs for clinical practice/clinical ethics. However, in the absence of national regulation, there is an urgent need for local promotion of ECs for clinical ethics or, alternatively, training for members of ECs with multiple functions, so that ethically sensitive issues such as those touched upon by law n. 219/2017 are not disregarded by these bodies.

## Fundings

Support for this study was provided by the Italian Ministry of Health with Ricerca Corrente and 5 × 1000 funds (2019) devolved to IRCCS Istituto Centro San Giovanni di Dio Fatebenefratelli.

## Supplementary Information

**Additional file 1**. Questionnaire (Italian version and English transaltion).

**Additional file 2**. Dataset with responses to the questionnaire (Italian).

**Additional file 3**. Dateset with responses to the questionnaire (English transaltion).

## Data Availability

The questionnaire used for the survey is available as Additional file 1 in the original Italian version and English translation. The dataset with responses to the questionnaire is available as Additional files 2, 3.

## Abbreviations

AIFA: Italian Medicine Agency
CNB: Italian National Bioethics Committee
EC: Ethics Committee
IRCCS: Italian Institute for Research and Care
MoH: Italian Ministry of Health
