# Supplementary material for: Italian law n. 219/2017 on consent and advance directives: survey among Ethics Committees on their involvement and possible role
Source: BMC Med Ethics. 2022 Nov 16;23:114. doi: 10.1186/s12910-022-00858-w (PMC9670375; doi:10.1186/s12910-022-00858-w)
Supplement: Supplementary file 1 — Additional file 1. Questionnaire (Italian version and English transaltion). [file 12910_2022_858_MOESM1_ESM.docx]

**Questionario**

* Campo obbligatorio

1) Avete avuto occasione di discutere in Comitato Etico (CE) la legge 219/2017? *

Sì Passa alla domanda 3

No Passa alla domanda 2

2) Se non avete discusso, perché? [è possibile più di una risposta] *

Seleziona tutte le voci applicabili

Non rientra nei compiti istituzionali del nostro CE

Non ci è stato richiesto dai professionisti della salute

Non ci è stato richiesto dalla/e struttura/e che fanno riferimento al CE

Per mancanza di tempo

Altro: …………………………………………………………………………………………………………………………………………………………

Passa alla domanda 9

3) In che modo avete discusso? [è possibile più di una risposta] *

Seleziona tutte le voci applicabili

In termini di riflessione teorica spontanea

Su richiesta di analisi di casi clinici specifici

Come risposta a quesiti dei professionisti della salute

Come risposta a quesiti della/e struttura/e che fanno riferimento al CE

All’interno di iniziative formative per i membri CE

All'interno di iniziative formative per gli operatori sanitari organizzate spontaneamente dal CE

All'interno di iniziative formative per gli operatori sanitari organizzate su incarico della/e struttura/e che fanno riferimento al CE

All'interno di iniziative formative che hanno coinvolto il pubblico organizzate spontaneamente dal CE

All'interno di iniziative formative che hanno coinvolto il pubblico organizzate su incarico della/e struttura/e che fanno riferimento al CE

Altro: …………………………………………………………………………………………………………………………………………………………

4) Quale aspetto della legge avete discusso? [è possibile più di una risposta] *

Seleziona tutte le voci applicabili

Consenso informato (Articolo 1; ad eccezione dei commi 8-10 relativi agli obblighi organizzativi e di formazione)

Obblighi organizzativi e di formazione (Articolo 1, commi 8-10)

Terapia del dolore, divieto di ostinazione irragionevole delle cure e dignità nella fase finale della vita (Articolo 2)

Minori (Articolo 3)

Incapaci (Articolo 3)

Disposizioni anticipate di trattamento (Articolo 4)

Pianificazione condivisa delle cure (Articolo 5)

5) Avete prodotto uno o più documenti? Es. pareri, linee di indirizzo... *

Sì

No

6) Se sì, sono disponibili? [Indicare dove è possibile reperirli/chi contattare per riceverli]

7) Avete ritenuto necessario rivedere i documenti già prodotti alla luce della normativa sopravvenuta? *

Sì

No

Il CE non aveva documenti pregressi

8) Se sì, sono disponibili? [Indicare dove è possibile reperirli/chi contattare per riceverli]

9) La/le struttura/e che fanno riferimento al vostro CE hanno prodotto procedure/linee di indirizzo/altri documenti in merito alla legge 219/2017? *

Sì

No

Non so

10) Se sì,

In collaborazione con il CE

Senza la collaborazione del CE

11) Sono disponibili? [Indicare dove è possibile reperirli/chi contattare per riceverli]

12) La/le struttura/e che fanno riferimento al vostro CE hanno ritenuto di dover rivedere procedure/linee di indirizzo/altri documenti alla luce della normativa sopravvenuta? *

Sì

No

Non so

13) Se si,

In collaborazione con il CE

Senza la collaborazione del CE

14) Sono disponibili? [Indicare dove è possibile reperirli/chi contattare per riceverli]

15) Secondo la sua esperienza di membro di CE, un coinvolgimento dei CE nell’applicazione della legge (pur in assenza di riferimento esplicito ai CE nel testo di legge) sarebbe:

assolutamente inopportuno Passa alla domanda 16

inopportuno Passa alla domanda 16

né inopportuno né opportuno Passa alla domanda 17

opportuno Passa alla domanda 18

assolutamente opportuno Passa alla domanda 18

16) Se assolutamente inopportuno/inopportuno, perché?

Passa alla domanda 20

17) Se né inopportuno né opportuno, perché?

Passa alla domanda 20

18) Relativamente a quali aspetti sarebbe opportuno un coinvolgimento dei CE?

a) Articolo 1: Consenso informato – ad eccezione degli aspetti relativi agli obblighi organizzativi e di formazione di cui ai commi 8-10 *

assolutamente in disaccordo

in disaccordo

né d'accordo né in disaccordo

d'accordo

assolutamente d'accordo

b) Articolo 1: Consenso informato – aspetti relativi agli obblighi organizzativi e di formazione di cui ai commi 8-10 *

assolutamente in disaccordo

in disaccordo

né d'accordo né in disaccordo

d'accordo

assolutamente d'accordo

c) Articolo 2: Terapia del dolore, divieto di ostinazione irragionevole delle cure e dignità nella fase finale della vita *

assolutamente in disaccordo

in disaccordo

né d'accordo né in disaccordo

d'accordo

assolutamente d'accordo

d) Articolo 3: Minori e incapaci – aspetti relativi ai Minori *

assolutamente in disaccordo

in disaccordo

né d'accordo né in disaccordo

d'accordo

assolutamente d'accordo

e) Articolo 3: Minori e incapaci - aspetti relativi agli Incapaci *

assolutamente in disaccordo

in disaccordo

né d'accordo né in disaccordo

d'accordo

assolutamente d'accordo

f) Articolo 4: Disposizioni anticipate di trattamento *

assolutamente in disaccordo

in disaccordo

né d'accordo né in disaccordo

d'accordo

assolutamente d'accordo

g) Articolo 5: Pianificazione condivisa delle cure *

assolutamente in disaccordo

in disaccordo

né d'accordo né in disaccordo

d'accordo

assolutamente d'accordo

19) Quale potrebbe essere il contributo dei CE relativamente agli aspetti sopra citati?

a) Articolo 1: Consenso informato - ad eccezione degli aspetti relativi agli obblighi organizzativi e di formazione di cui ai commi 8-10

b) Articolo 1: Consenso informato - aspetti relativi agli obblighi organizzativi e di formazione di cui ai commi 8-10

c) Articolo 2: Terapia del dolore, divieto di ostinazione irragionevole delle cure e dignità nella fase finale della vita

d) Articolo 3: Minori e incapaci – aspetti relativi ai Minori

e) Articolo 3: Minori e incapaci - aspetti relativi agli Incapaci

f) Articolo 4: Disposizioni anticipate di trattamento

g) Articolo 5: Pianificazione condivisa delle cure

h) Altri aspetti: [indicare]

20) Secondo la sua esperienza di membro di CE di un IRCCS di neuroscienze e neuroriabilitazione, esistono particolari situazioni cliniche e/o patologie per le quali potrebbe essere maggiormente utile un coinvolgimento dei CE territoriali nell’applicazione della legge? *

Sì

No

Non so

21) Se sì, quali:

22) Eventuali osservazioni aggiuntive:

**Questionnaire**

* Required answer

1) Did you have the opportunity to discuss Law n. 219/2017 in the Ethics Committee (EC)? *

Yes Skip to question 3

No Skip to question 2

2) If you did not discuss, why? [more than one answer is possible] *

Select all applicable items

Not within the institutional tasks of our EC

We were not requested by health professionals

We were not requested by the facility(ies) that refer to the EC

Due to lack of time

Other: ………………………………………………………………………………………………………………………………………………………..

Skip to question 9

3) How did you discuss? [more than one answer is possible] *

Select all applicable items

In terms of spontaneous theoretical reflection

Upon request for analysis of specific clinical cases

As a response to questions from health professionals

As a response to questions from the facility(ies) that refer to the EC

Within training initiatives for EC members

Within training initiatives for health professionals organized spontaneously by the EC

Within training initiatives for health professionals organized by the EC on behalf of the facility(ies) that refer to the EC

Within training initiatives that involved the public organized spontaneously by the EC

Within training initiatives that involved the public organized by the EC on behalf of the facility(ies) that refer to the EC

Other: ………………………………………………………………………………………………………………………………………………………

4) What aspect of the Law did you discuss? [more than one answer is possible] *

Select all applicable items

Informed consent (Article 1; except paragraphs 8 to 10 on organizational and training obligations)

Organizational and training obligations (Article 1, paragraphs 8 to 10)

Pain therapy, prohibition of unreasonable obstinacy in treatment and dignity in the end of life (Article 2)

Minors (Article 3)

Incapacitated adults (Article 3)

Advance directives (Article 4)

Shared care planning (Article 5)

5) Did you produce one or more documents? E.g. opinions, guidelines…*

Yes

No

6) If yes, are these documents available? [indicate where they can be found/who to contact to receive them]

7) Did you consider it necessary to review documents you already produced in light of the new regulation? *

Yes

No

The EC did not have previous documents

8) If yes, are these documents available? [indicate where they can be found/who to contact to receive them]

9) Did the facility(ies) that refer to your EC produce procedures/guidelines/other documents regarding Law n. 219/2017? *

Yes

No

I don’t know

10) If yes,

With the collaboration of the EC

Without the collaboration of the EC

11) Are these documents available? [indicate where they can be found/who to contact to receive them]

12) Did the facility(ies) that refer to the EC consider it necessary to review procedures/guidelines/other documents in light of the new regulation? *

Yes

No

I don’t know

13) If yes,

With the collaboration of the EC

Without the collaboration of the EC

14) Are these documents available? [indicate where they can be found/who to contact to receive them]

15) In your experience as an EC member, an involvement of the ECs in the Law implementation (even in the absence of explicit reference to ECs in the text of the Law) would be: *

absolutely inappropriate Skip to question 16

inappropriate Skip to question 16

neither inappropriate nor appropriate Skip to question 17

appropriate Skip to question 18

absolutely appropriate Skip to question 18

16) If absolutely inappropriate/inappropriate, why?

Skip to question 20

17) If neither inappropriate nor appropriate, why?

Skip to question 20

18) In relation to what aspects of the Law would EC involvement be appropriate?

a) Article 1: Informed consent - except for aspects related to organizational and training obligations in paragraphs 8 to 10 *

absolutely disagree

disagree

neither agree nor disagree

agree

absolutely agree

b) Article 1: Informed consent – aspects related to organizational and training obligations in paragraphs 8 to 10 *

absolutely disagree

disagree

neither agree nor disagree

agree

absolutely agree

c) Article 2: Pain therapy, prohibition of unreasonable obstinacy in treatment and dignity in the end of life*

absolutely disagree

disagree

neither agree nor disagree

agree

absolutely agree

d) Article 3: Minors and incapacitated adults - aspects related to Minors *

absolutely disagree

disagree

neither agree nor disagree

agree

absolutely agree

e) Article 3: Minors and incapacitated adults - aspects related to Incapacitated adults *

absolutely disagree

disagree

neither agree nor disagree

agree

absolutely agree

f) Article 4: Advance directives *

absolutely disagree

disagree

neither agree nor disagree

agree

absolutely agree

g) Article 5: Shared care planning *

absolutely disagree

disagree

neither agree nor disagree

agree

absolutely agree

19) What could be the contribution of the ECs regarding the aforementioned aspects?

a) Article 1: Informed consent – except for aspects related to organizational and training obligations in paragraphs 8 to 10

b) Article 1: Informed consent – aspects related to organizational and training obligations in paragraphs 8 to 10

c) Article 2: Pain therapy, prohibition of unreasonable obstinacy in treatment and dignity in the end of life

d) Article 3: Minors and incapacitated adults - aspects related to Minors

e) Article 3: Minors and incapacitated adults - aspects related to Incapacitated adults

f) Article 4: Advance directives

g) Article 5: Shared care planning

h) Other aspects: [indicate]

20) In your experience as an EC member of a neuroscience and neurorehabilitation institute, are there particular clinical situations and/or pathologies for which an ECs involvement in Law implementation might be most useful? *

Yes

No

I don’t know

21) If yes, which ones?

22) Additional comments (if any):
